# DsbA-L safeguards T cell mitochondrial redox homeostasis to restrain Th17 differentiation and colitis

**DOI:** 10.1038/s41419-026-08978-6

**Published:** 2026-06-13

**Authors:** Jiangming Deng, Liwen Wang, Bilian Liu, Jing Wang, Qingxin Li, Yi Deng, Long Yan, Hairong Luo, Wen Meng, Feng Liu

**Affiliations:** 1 National Clinical Research Center for Metabolic Diseases, The Second Xiangya Hospital of Central South University, Changsha, Hunan China; 2 Metabolic Syndrome Research Center, The Second Xiangya Hospital of Central South University, Changsha, Hunan China; 3 Department of Endocrinology, Endocrinology Research Center, Xiangya Hospital of Central South University, Changsha, Hunan China; 4 Department of Oncology, The Second Xiangya Hospital of Central South University, Changsha, Hunan China

**Keywords:** Gastrointestinal diseases, Inflammation

## Abstract

Pathogenic Th17 accumulation drives intestinal inflammation in inflammatory bowel disease (IBD), yet the metabolic mechanisms directing Th17 polarization remain incompletely understood. Here, we identify the mitochondrial matrix protein DsbA-L serves as a critical, cell-intrinsic metabolic checkpoint for Th17 differentiation and intestinal immune homeostasis. In mouse models, dextran sulfate sodium (DSS)-induced colitis downregulated DsbA-L expression and disrupted mitochondrial redox balance in intestinal CD4⁺ T cells. T cell-specific DsbA-L deletion exacerbated colitis and selectively promotes pathogenic Th17 differentiation by increasing mitochondrial reactive oxygen species (mtROS). Pharmacological mtROS scavenging reverses the Th17 bias, establishing mitochondrial redox imbalance as a causal driver of pathogenic polarization. Furthermore, DsbA-L is essential for the therapeutic efficacy of the PPARγ agonist rosiglitazone in colitis. Collectively, our findings position DsbA-L-mediated mitochondrial redox balance as a key regulator of Th17 pathogenicity and intestinal inflammation, offering new conceptual and therapeutic insights for precision intervention in IBD.

**Figure:**
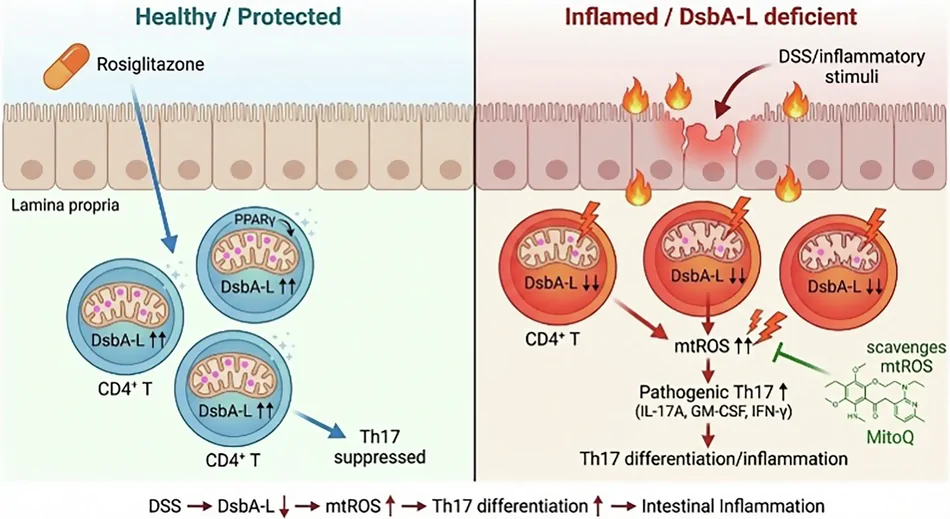

## Introduction

Pathogenic CD4⁺ T cell responses, particularly those driven by Th17 cells, are central to the pathogenesis of inflammatory bowel disease (IBD) [1–3]. While genetic and cytokine-based mechanisms regulating Th17 differentiation have been extensively studied, emerging evidence suggests that T cell fate decisions are tightly constrained by cellular metabolic state rather than transcriptional cues alone. How metabolic checkpoints within T cells shape Th17 polarization in the inflamed intestine, however, remains poorly defined.

The intestinal tract faces constant immune challenges from dietary factors and antigens, making it a key site for immune regulation and pathology [4]. Inflammatory CD4⁺ T cells, particularly Th17 cells, drive intestinal inflammation in IBD by producing interleukin-17 and disrupting the epithelial barrier, while Treg cells mitigate this by secreting IL-10 [1, 5, 6]. Th17-associated pathways are strongly linked to IBD susceptibility and s severity [7, 8]. Yet the mechanisms governing Th17 differentiation and pathogenicity in the intestinal microenvironment remain unclear.

Recent evidence highlights the tight interplay between immune cell metabolism and function. Mitochondria act as signaling hubs in adaptive immunity, integrating metabolic demands and redox signals to guide T cell differentiation [9–11]. In CD4⁺ T cells, mitochondrial reactive oxygen species (mtROS) actively promote Th17 lineage commitment [12, 13]. suggesting that mitochondrial redox homeostasis may serve as a critical checkpoint for Th17 differentiation during intestinal inflammation.

Disulfide bond A oxidoreductase-like protein (DsbA-L), a mitochondrial chaperone known for preserving mitochondrial integrity and redox balance in metabolic tissues [14, 15]. Has recently been implicated in T cell metabolic regulation [16]. However, it functions as a cell-intrinsic mitochondrial checkpoint for T cell fate, and its impact on Th17 differentiation and intestinal inflammation remains to be defined.

Here, we identify T cell-intrinsic DsbA-L as a mitochondrial redox checkpoint that restrains pathogenic Th17 differentiation during colitis. Using genetic, metabolic, and pharmacological approaches, we demonstrate that loss of DsbA-L disrupts mitochondrial redox homeostasis, amplifies Th17 responses, and exacerbates DSS-induced colitis, while being required for the full protective effects of PPARγ agonism. Together, our findings establish a mechanistic link between mitochondrial fitness, T cell fate control, and intestinal inflammation.

## Results

### DSS-induced colitis impairs CD4^+^ T cell mitochondrial integrity in association with DsbA-L downregulation

**Fig. 1:**
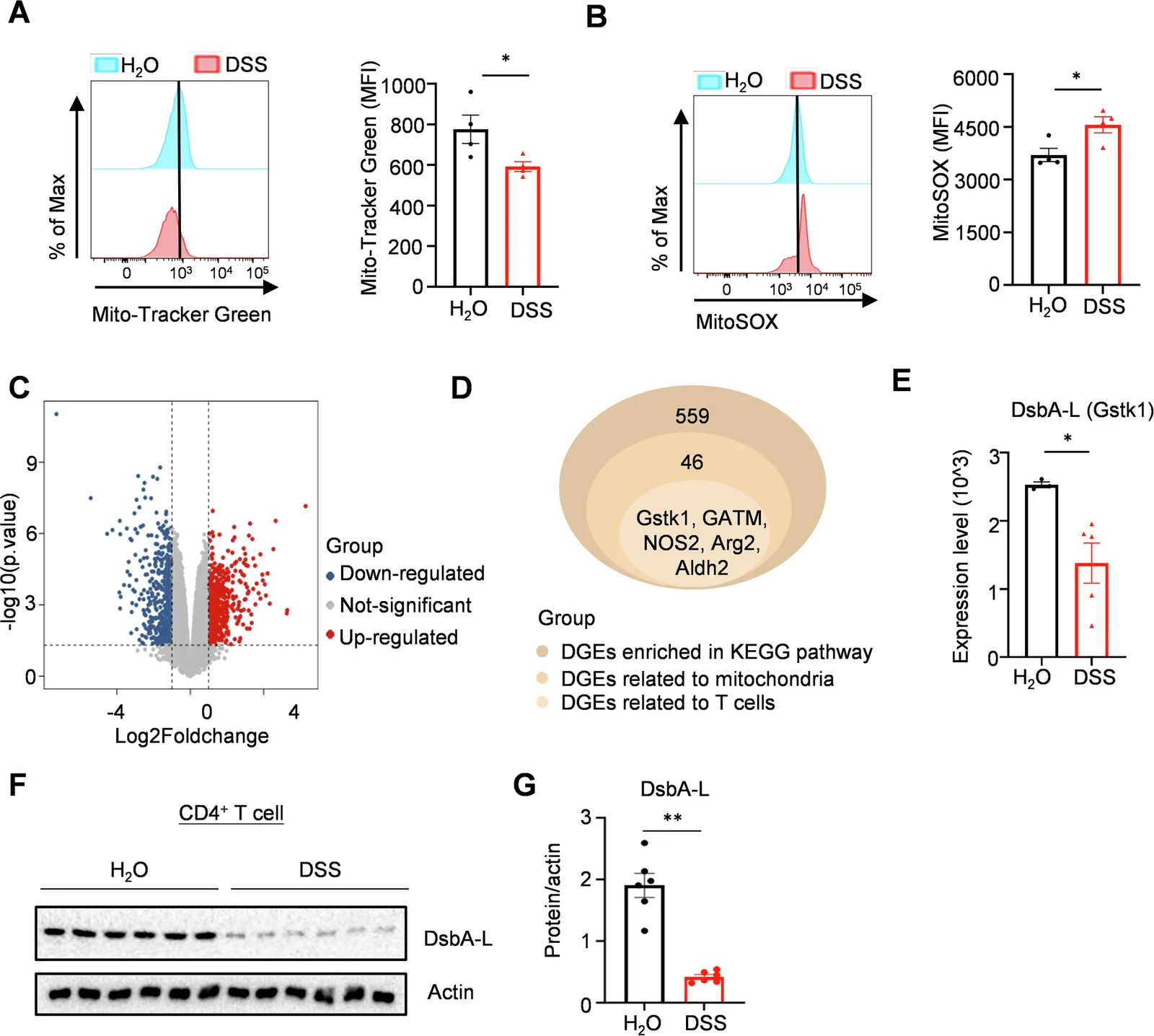
DSS-Induced Colitis Is Associated with Reduced Mitochondrial Mass and Increased mtROS in Intestinal CD4^+^ T Cells. Mitochondrial mass (**A**) and mitochondrial ROS (**B**) of CD3^+^CD4^+^T cells in colonic lamina propria from DSS-induced colitis was analyzed by Mito-Tracker Green and MitoSOX staining respectively (*n* = 4 biological replicates per group). **C** Volcano plots of differentially expressed genes (DEGs). **D** The Venn diagram showing the strategy to identify T cell mitochondrial function -related factors. **E** Violin plots of trans-log2 transferred gene expression of Gstk1 genes in colon tissue. **F**, **G** Representative immunoblots and quantification of DsbA-L expression of colonic isolated CD4^+^ T cells from DSS-induced colitis (*n* = 6 biological replicates per group). Data shown are representative of three independent experiments. All data are presented as mean ± SEM. Statistical significance was assessed by unpaired Student’s *t* test (**A**, **B**, **E**, **G**). *P < 0.05, **P < 0.01.

To determine whether mitochondrial dysfunction in CD4^+^ T cell is associated with intestinal inflammation, we evaluated mitochondrial mass in CD4^+^ T cells isolated from the colonic lamina propria of DSS- or H_2_O-treated mice. DSS treatment led to a marked reduction in mitochondrial mass, a surrogate for mitochondrial integrity [17]. and was accompanied by typical colitis symptoms, including weight loss (Suppl. Fig. 1 and Fig. 1A), rectal bleeding, and colon shortening (Suppl. Fig. 2A–C). Flow cytometry further revealed a significant increase in mtROS in colonic lamina propria cells from DSS-treated mice (Fig. 1B). These observations indicate that DSS-induced colitis disrupts mitochondrial homeostasis in intestinal CD4⁺ T cells.

To elucidate the underlying mechanism, we analyzed a published Gene Expression Omnibus (GEO) database (GSE73424) from DSS-induced colitis mouse colon tissues [18]. Volcano plot analysis identified 1092 differentially expressed genes (DEGs) using a ≥ 2-fold change and *P* < 0.05 as cutoff criteria (Fig. 1C), with 559 enriched in mitochondrial-related Kyoto Encyclopedia of Genes and Genomes (KEGG) pathways and 46 directly linked to mitochondrial function (Supplementary Fig. 2D and Fig. 1D). Of these, five genes, *Gstk1, Gatm, Nos2, Arg2*, and *Aldh2*, encoding DsbA-L, glycine amidinotransferase, nitric oxide synthase 2, arginase 2, and aldehyde dehydrogenase 2, respectively, were relevant to T cell biology (Fig. 1D). Notably, the mRNA expression of DsbA-L, a known regulator of T cell mitochondrial function [16]. was significantly reduced at both the mRNA in colon tissues (Fig. 1E) and protein (Fig. 1F, G) levels in CD4⁺ T cells following DSS treatment, suggesting that DsbA-L downregulation may drive mitochondrial defects and increased mtROS in colitis.

To establish clinical relevance, we analyzed human IBD datasets (GSE179285, GSE66407) and found that expression of GSTK1, the gene encoding DsbA-L, was significantly reduced in colonic biopsies from patients with ulcerative colitis (UC) and Crohn’s disease (CD) compared to healthy controls (Supplementary Fig. 3). These results support and extend our findings from the mouse model.

### T cell-intrinsic DsbA-L protects against DSS-induced colitis

**Fig. 2:**
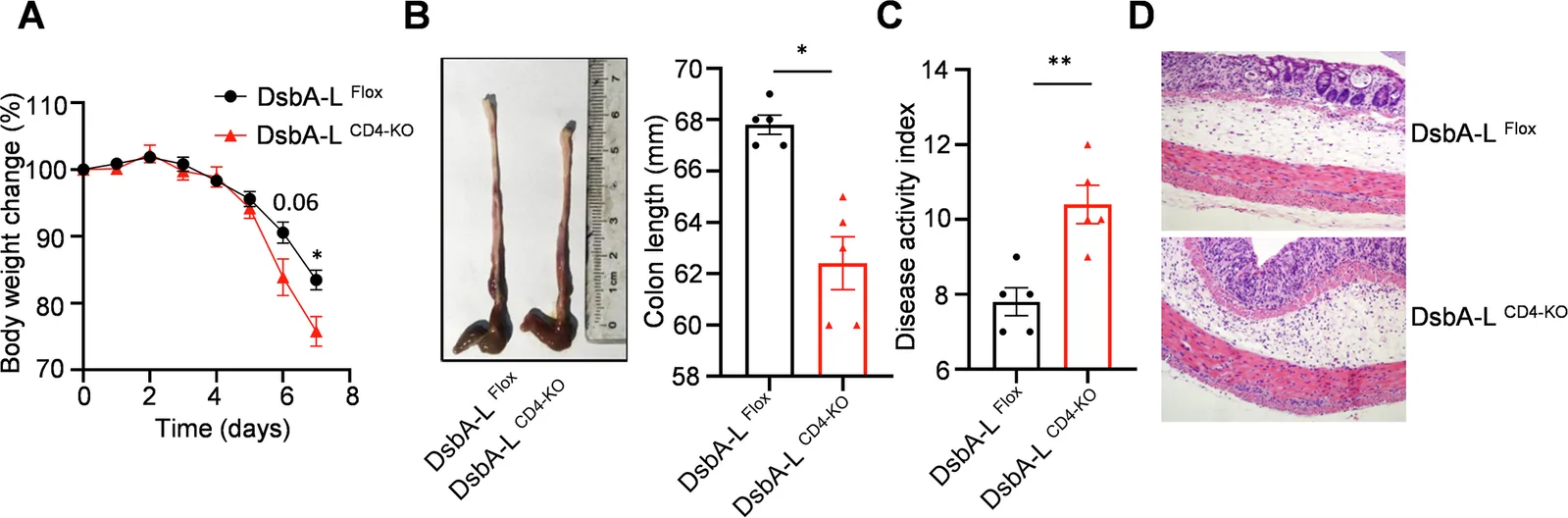
DsbA-L deficiency in T cells aggravates DSS-induced colitis. DsbA-L^CD4-KO^ and control littermates (DsbA-L^Flox^) were given 4% DSS for 7 days (*n* = 5 biological replicates per group). Loss of basal body weight (**A**), colon length (**B**), and disease activity index (**C**) were measured. (**D**) histology images of colon sections. Data shown are representative of three independent experiments. All data are presented as mean ± SEM. Statistical significance was assessed by two-way ANOVA (**A**) or unpaired Student’s *t* test (**B**, **C**). *P < 0.05; **P < 0.01.

To determine the role of T cell DsbA-L in T cells during intestinal inflammation, we utilized T cell-specific DsbA-L knockout mice (DsbA-L^CD4-KO^) as previously described [16]. Following DSS treatment, DsbA-L^CD4-KO^ mice exhibited significantly greater body-weight loss (Fig. 2A), reduced colon length (Fig. 2B), and higher disease activity index (DAI) scores (Fig. 2C) compared to floxed controls. Histologic analysis showed exacerbated colitis in DSS-treated DsbA-L^CD4-KO^ mice, characterized by severe villous damage, extensive crypt loss, and pronounced lymphocytic infiltration (Fig. 2D). Together, these results indicate that the absence of DsbA-L in CD4⁺ T cells markedly increases susceptibility to DSS-induced colitis, highlighting the protective role of T cell-intrinsic DsbA-L in maintaining intestinal homeostasis.

### DsbA-L cell-autonomously restrains Th17 differentiation in vitro

**Fig. 3:**
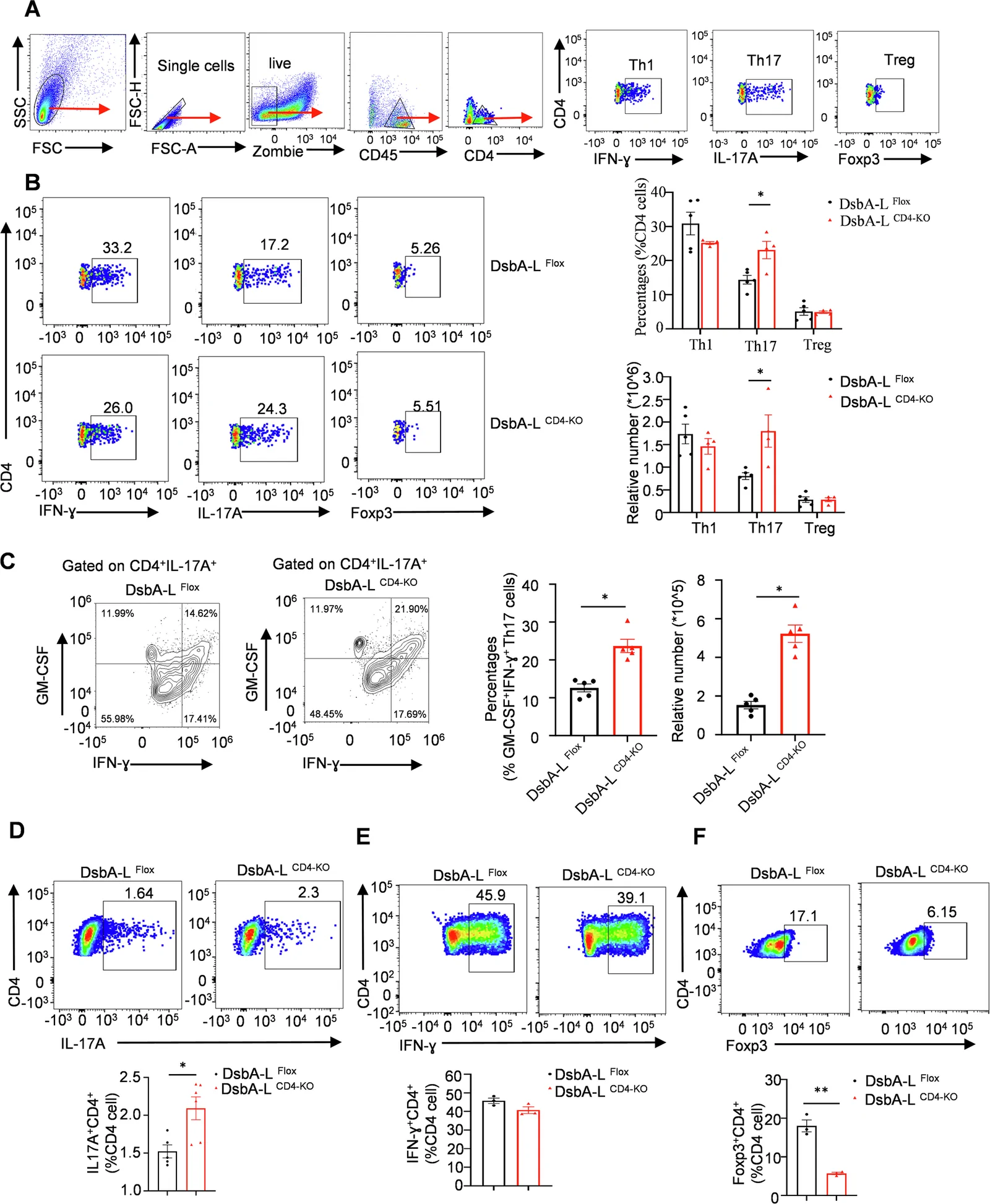
DsbA-L deficiency in T cells promotes Th17 cells differentiation. DsbA-L^CD4-KO^ mice and control littermates were fed 4% DSS for 7 days (*n* = 4–5 biological replicates per group). **A** Gating strategies of T cell subsets in colonic lamia propria. **B** Representative FACS plots of T cells subsets (left) and quantification of the frequencies of T cell subset (right). **C** Representative FACS plots and frequency of GM-CSF^+^IFN-γ^+^ Th17 cells. Representative FACS plots and frequency of Th17 cells (IL-17A^+^CD4^+^ T cells) (**D**), Th1 cells (IFN-γ^+^CD4^+^ T cells). (**E**) and Treg cells (Foxp3^+^CD4^+^ T cells) (**F**) of CD4^+^CD25^-^CD62L^+^ naive T cells culture in vitro. CD4^+^CD25^-^CD62L^+^ naive T cells were cultured in complete DMEM containing specific polarization cytokines. Representative FACS plots of T cells subsets (up) and quantification of the frequencies of T cell subset (down). All data are presented as mean ± SEM. Statistical significance was assessed by unpaired Student’s *t* test (**B**–**E**). *P < 0.05; **P < 0.01.

An increased accumulation of Th1 and Th17 cells within intestinal tissues is a well-established driver of gut barrier dysfunction and intestinal inflammation [1]. Flow cytometry analysis revealed that, following DSS treatment, the proportion of Th17 cells, but not Th1 and Treg cells, was significantly increased in the lamina propria of DsbA-L^CD4-KO^ mice compared to floxed control mice (Fig. 3A, B). Notably, DsbA-L deficiency did not affect T cell subset distribution in the spleen or mesenteric lymph nodes (Suppl. Fig. 4A, B), indicating a gut-specific effect. Given the functional heterogeneity of Th17 cells, further analysis showed that DsbA-L deficiency led to a higher proportion of pathogenic GM-CSF⁺ IFN-γ⁺ Th17 cells in the colon (Fig. 3C). These findings suggest that loss of DsbA-L in T cells preferentially promotes expansion and pathogenic polarization of intestinal Th17 cells during inflammation.

To determine whether DsbA-L directly regulates Th17 cell differentiation, we cultured naïve CD4⁺CD25⁻CD62L⁺ T cells from DsbA-L-deficient and wild-type mice under Th17-polarizing conditions. DsbA-L-deficient T cells exhibited a significantly greater proportion of Th17 cells than wild-type controls (Fig. 3D), indicating that DsbA-L cell-autonomously restrains Th17 differentiation. In line with in vivo results (Fig. 3B), DsbA-L deficiency did not affect Th1 differentiation in vitro (Fig. 3E). However, DsbA-L knockout slightly reduced Treg differentiation (Fig. 3F), suggesting that additional compensatory mechanisms in vivo may help preserve Treg homeostasis in the absence of DsbA-L.

### Mitochondrial ROS mediates enhanced Th17 differentiation in DsbA-L–Deficient T cells

**Fig. 4:**
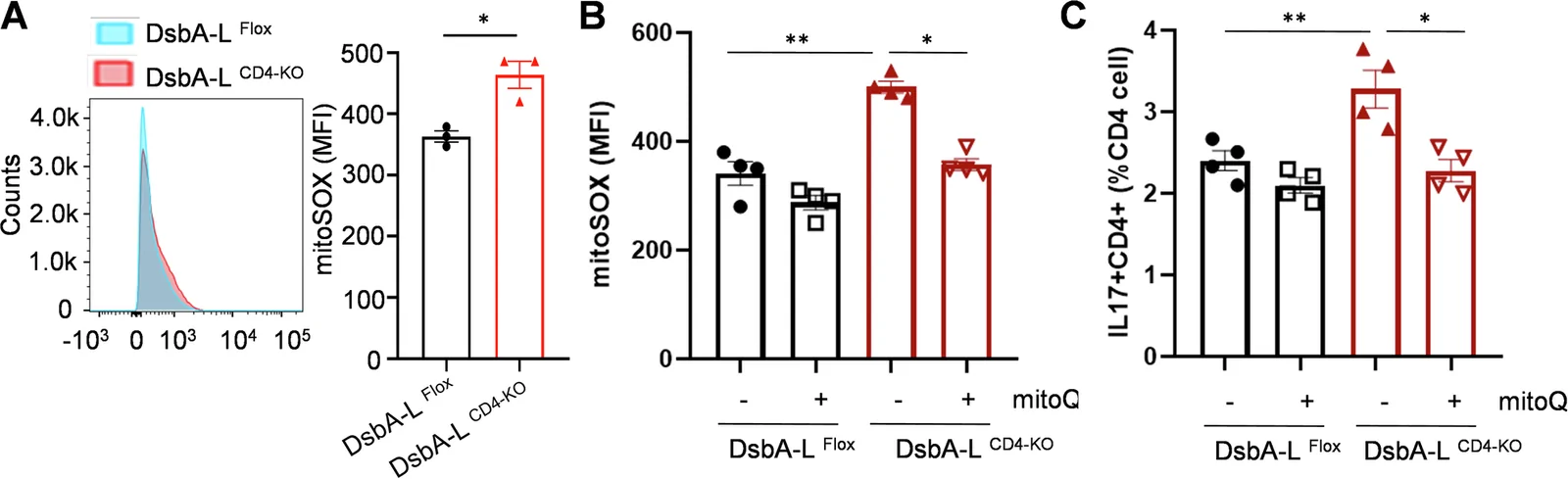
DsbA-L deficiency in T cells promotes Th17 cells differentiation by increased mitochondrial ROS. **A**, **B** Mitochondrial MitoSOX (MFI) intensity T-cells of DsbA-L^CD4-KO^ and control littermates (DsbA-L^Flox^) treated with mitoQ or control (*n* = 3–4/group, 3 technical replicate of 3-4 biological replicates per group). **C** Th17 cells differentiation of DsbA-L^CD4-KO^ and control littermates treated with mitoQ or control (*n* = 4/group, 3 technical replicate of 4 biological replicates per group). Data shown are representative of three independent experiments. All data are presented as mean ± SEM. Statistical significance was assessed by unpaired Student’s t test (**A**) or two-way ANOVA (**B**, **C**). *P < 0.05; **P < 0.01.

Multiple mechanisms have been implicated in the regulation of Th17 cells differentiation, including STAT3-dependent expression of RORγt [19]. cytokine such as IL-6, IL-21, and IL-23-triggered signaling [20]. and altered mitochondrial ROS signaling [12, 13]. Given that loss of DsbA-L elevated mitochondrial ROS levels in hepatocytes [14]. We measured mtROS levels in DsbA-L deficient CD4^+^ cells. Depletion of DsbA-L significantly increased mtROS levels in T cells (Fig. 4A). To test whether increased mtROS drives the enhanced Th17 differentiation observed in DsbA-L-deficient T cells, naïve T cells from DsbA-L^CD4-KO^ or floxed control mice were treated with the mitochondria-targeted antioxidant mitoQ. MitoQ treatment effectively reduced mtROS levels (Fig. 4B) and, accordingly, diminished the heightened Th17 differentiation caused by DsbA-L deficiency (Fig. 4C). These findings identify mtROS as a key mediator linking DsbA-L loss to increased Th17 differentiation.

### Rosiglitazone attenuates colitis with a critical contribution from T Cell-intrinsic DsbA-L

**Fig. 5:**
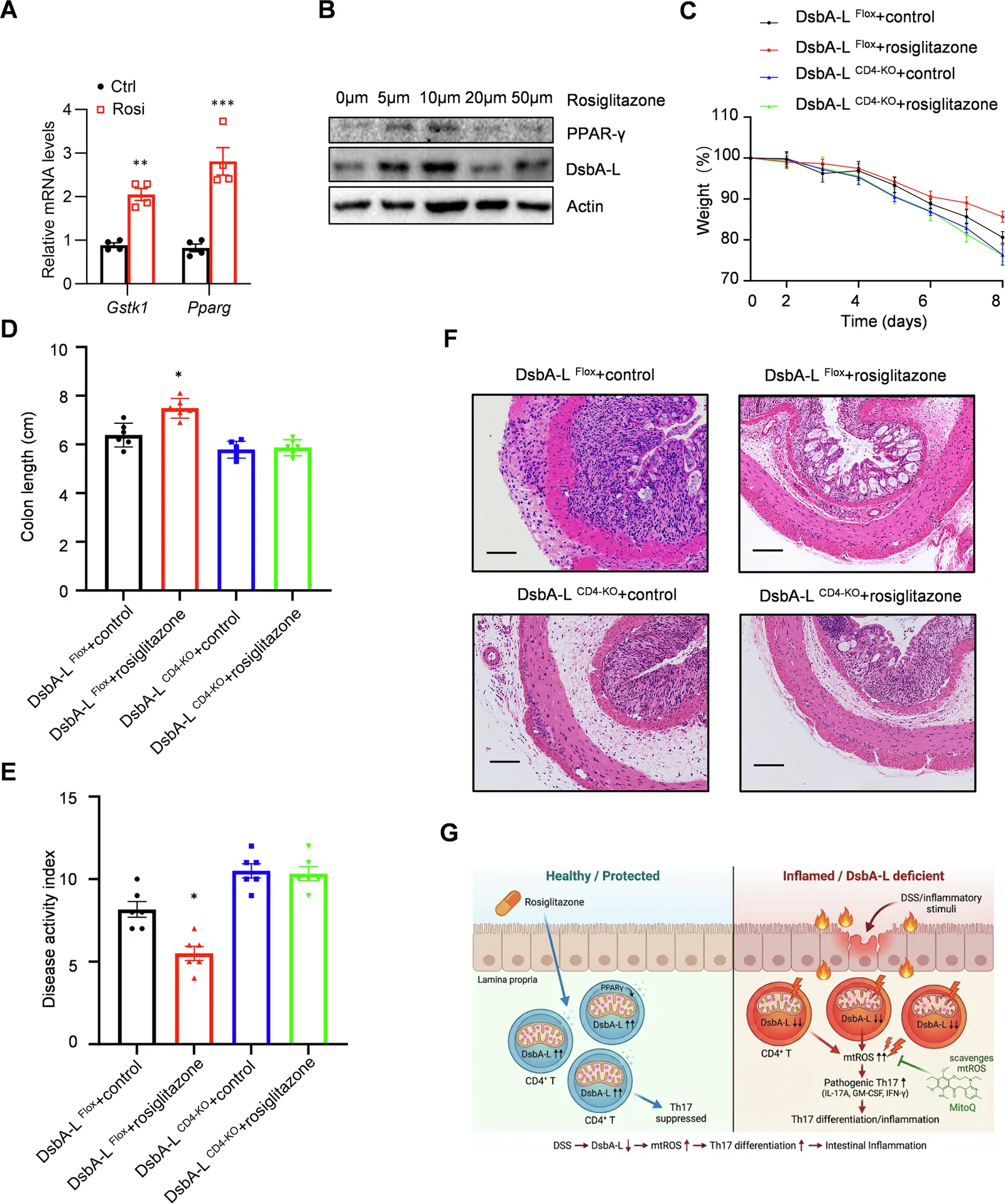
T cell DsbA-L contributes to rosiglitazone-ameliorated DSS-induced colitis. **A** Relative mRNA of *Gstk1* and *Pparg* in Rosiglitazone-treated T cell (*n* = 4/group, 3 technical replicate of 4 biological replicates per group). **B** Representative immunoblots of DsbA-L and PPAR-γ (protein) in Rosiglitazone-treated T cell (Representative of 3 biological replicates). **C**–**F** DsbA-L^CD4-KO^ and control littermates (DsbA-L^Flox^) were given 4% DSS in water and Rosiglitazone (5 mg/kg/day) or control by gavage (*n* = 6 biological replicates per group). Loss of basal body weight (**C**), colon length (**D**), and disease activity index (**E**) were measured. (**F**) histology images of colon sections (bar:100 µm). **G** Schematic summary. Data shown are representative of three independent experiments. All data are presented as mean ± SEM. Statistical significance was assessed by unpaired Student’s t test (**A**) or two-way ANOVA (**C**–**E**). *P < 0.05; **P < 0.01; ***P < 0.001.

Treating T cells with rosiglitazone, a PPARγ agonist previously shown to induce DsbA-L expression in adipocytes [21]. robustly increased DsbA-L mRNA (Fig. 5A) and protein levels (Fig. 5B). Since oral rosiglitazone has been reported to protect against experimental colitis [22]. We investigated whether this effect is mediated by upregulation of DsbA-L in T cells. Rosiglitazone administration significantly ameliorated DSS-induced colitis in floxed control mice, as reflected by reduced body weight loss (Fig. 5C), elongated colon length (Fig. 5D), lower disease activity index (DAI) scores (Fig. 5E), and improved histopathology (Fig. 5F). However, these protective effects were partially reduced in DSS-treated DsbA-L^CD4-KO^ mice (Fig. 5C–F). Together, these results indicate that the therapeutic benefit of rosiglitazone against colitis is at least partially dependent on T cell-intrinsic DsbA-L, though other cell types such as macrophages or intestinal epithelial cells, may also contribute to its protective action.

## Discussion

Pathogenic Th17 cell infiltration and CD4⁺ T cell–driven immune responses are central to intestinal inflammation and barrier dysfunction in IBD [1–3]. However, the upstream mechanisms selectively promoting Th17 differentiation in the inflamed gut remain poorly defined. Here, we reveal the mitochondrial chaperone DsbA-L as a key regulator of T cell fate in intestinal inflammation. DsbA-L expression is markedly reduced in CD4⁺ T cells during DSS-induced colitis, and its deficiency exacerbates disease severity, elevates mtROS, and selectively promotes expansion of pathogenic Th17 cells, characterized by increased IL-17A, GM-CSF, and IFN-γ production, which are closely linked to tissue injury in IBD [23–25].

Mechanistically, our findings underscore mitochondrial redox dysregulation as a pivotal link between DsbA-L loss and Th17 bias. Consistent with prior reports in metabolic tissues [14, 15]. DsbA-L deficiency in CD4⁺ T cells dramatically increases mtROS, and pharmacological scavenging of mtROS restores balanced Th17 differentiation [12, 13]. This highlights mitochondrial redox control as a central metabolic checkpoint dictating T cell fate and inflammatory outcomes in the intestine.

Beyond advancing our understanding of T cell immunometabolism, our findings reveal that the protective effects of PPARγ agonists in experimental colitis depend on T cell-intrinsic DsbA-L, extending the role of PPARγ beyond lipid metabolism and macrophage biology to include direct regulation of T cell mitochondrial redox balance [26–29]. This defines a novel PPARγ-DsbA-L-mitochondrial axis linking nuclear receptor signaling to immune regulation. Importantly, reduced GSTK1 (DsbA-L) expression in human IBD tissues supports the translational relevance of this pathway. Thus, selectively targeting DsbA-L or mitochondrial redox homeostasis in T cells may provide a precision therapeutic strategy for IBD, potentially minimizing the systemic side effects of broad PPARγ activation. Further single-cell or purified CD4⁺ T cell analyses in human IBD samples will help clarify cell-specific effects.

This study has several limitations. First, the DSS model primarily reflects acute, injury-driven colitis, which may not fully recapitulate the immune-driven pathology of other IBD models, such as spontaneous colitis [30, 31] or T cell-transfer models driven by adaptive immunity [32]. Future studies using these models are needed to determine whether the DsbA-L-mtROS-Th17 axis plays a similar role across diverse IBD subtypes and contexts. Second, our analyses of mitochondrial homeostasis focused on mitochondrial mass and mtROS levels, without comprehensive assessment of mitochondrial bioenergetics. Additional studies examining respiratory function and other aspects of mitochondrial biology will provide deeper mechanistic insight. Our previous study showed that DsbA-L deficiency impairs T cell mitochondrial function [16]. while the precise mechanism by which DsbA-L controls mitochondrial function was not fully addressed in this study. Finally, while we show that T cell-intrinsic DsbA-L is necessary for the full protective effect of rosiglitazone in colitis, PPARγ agonists also act on other cell types, including macrophages and epithelial cells [33, 34]. Thus, while T cell DsbA-L is a critical mediator, it may not fully account for all therapeutic effects of PPARγ activation.

In summary, our findings reveal mitochondrial redox regulation within T cells as a key determinant of Th17-driven immune responses during intestinal inflammation. By identifying DsbA-L as a T cell-specific metabolic checkpoint linking mitochondrial homeostasis to T cell fate, this study advances our understanding of adaptive immunity in inflamed tissues. More broadly, these results highlight mitochondrial redox control as a novel and actionable target for immune regulation, offering new conceptual and translational insights into the metabolic control of adaptive immunity in inflammatory bowel disease.

## Methods

### Mice

T cell-specific DsbA-L knockout mice (DsbA-L^CD4-KO^) were generated as described previously [16]. C57BL/6 mice were purchased from Hunan SJA Laboratory Animal Co., Ltd. (Changsha, China). All mice used for experiments were aged 8–12 weeks. Sample size was not predetermined, but we performed experiments with group sizes based on existing published literature of similar experiments [35, 36]. The samples used in this study were randomly assigned to control or experimental groups.

### DSS-induced colitis

To induce acute colitis, mice were administered 4% dextran sulfate sodium (DSS) (M.W. 36–50 kDa; Meilune; China) dissolved in water for seven days. Mouse body weight and stool consistency were checked daily. Disease severity index was calculated by assessing the extent of body weight decrease, stool consistency, and rectal hemorrhage as described previously [37]. Briefly, loss of body weight (0, none; 1, 1–5%; 2, 6–10%; 3, 11–18%; 4, >18%), stool consistency (0, normal; 1, soft but still formed; 2, soft; 3, very soft; 4, watery diarrhea), blood (0-1, negative hemoccult; 2, positive hemoccult; 3, blood traces in stool visible; 4, gross rectal bleeding). The control groups were treated with water. For rosiglitazone group, mice received rosiglitazone (5 mg/kg/day, gavage) from 7 days before the experiment to the experimental endpoint.

### Flow cytometry analysis

Single-cell suspensions were incubated with Fc block (anti-CD16/32, BioLegend, 101320) for 10 min at 4 °C to minimize non-specific binding, followed by surface marker staining by indicated antibody solutions for 30 min at 4 °C in dark. Following surface staining, intranuclear transcription factor staining was carried out with the Foxp3/Transcription Factor Staining Buffer Set according to the manufacturer’s instructions (eBioscience, CA, USA). Intracellular antibodies were diluted in 1x Permeabilization Buffer and incubated for 30 min at 4 °C. For intracellular cytokine staining, cells were stimulated with cell activation cocktail containing PMA (50 ng/ml, Beyotime, Shanghai, China), ionomycin (750 ng/ml, Beyotime, Shanghai, China), and Brefeldin A (10 μg/ml, Beyotime, Shanghai, China) for 6 h in vitro, and then harvested and fixed with the intracellular fixation/permeabilization using the Cytofix/Cytoperm kit (BD Biosciences). according to the manufacturer’s instructions. Cytokine antibodies were incubated for 30 min at 4 °C. Commercial antibodies used included Zombie NIR (BioLegend, 423102), APC-Cy7-CD45 (BioLegend, 103116), Percp/Cy5.5-CD3 (BioLegend, 100328), FITC-CD4 (BioLegend, 100510), PE-IL17A (BioLegend, 506903), Pacific Blue^™^-IFNγ (BioLegend, 505817), Alexa Fluor® 647-FOXP3 (BioLegend, 567462) and APC-GM-CSF (Invitrogen, Cat# 17-7331-82). For assessment of mitochondrial mass and membrane potential, colonic lamina propria cells were incubated with Zombie NIR, APC-Cy7-CD45, PE-Cy7-CD3 (BioLegend, 100220), Percp-CD4 (BioLegend,100434), 200 nM Mito-tracker green (Yeasen Biotechnology, 40742ES50), and 5 μM MitoSOX Red (Yeasen Biotechnology, 40778ES50) for 30 min at 37 °C. CD4⁺ T cells (CD3⁺CD4⁺) from the colonic lamina propria were then gated and analyzed. Cells were collected on a BD Canto II using the BD FACS Diva software. Stained cells were analyzed on BD FACSCalibur, and data were analyzed with FlowJo V10 software.

### Isolation of colonic lamina propria cell

Colonic lamina propria cell isolation was performed as previously described [38]. Briefly, colon tissues were freshly isolated, longitudinally opened, and washed with PBS. Intraepithelial lymphocytes (IEL) were depleted after incubating within PBS containing 15 mM HEPES and 5 mM EDTA in a 220-rpm shaking incubator for 30 min. The rest colon tissues were digested with type 4 collagenase (0.5 mg/ml, Sigma, USA) and DNase I (0.2 mg/ml, Sigma, USA) under vigorous shaking conditions. Isolated cell suspensions were used for flow cytometry analysis, or isolated colonic CD4^+^ T cells by using a mouse CD4^+^ T cell isolation kit (Miltenyi, Germany) as previously described [16].

### Cell culture

CD4^+^CD25^−^CD62L^+^ naive T cells purified by magnetic cell sorting (Miltenyi; Germany) from mouse spleens and peripheral lymph nodes were cultured at 1 × 10^6^ cells per well in 48-well plates with plate-bound 5 ug/ml anti-mouse-CD3 (BD Biosciences, San Jose, USA) and soluble 2 ug/mL anti-mouse-CD28 (Biolegend, San Diego, USA), and the polarizing cytokines: 5 ng/mL TGF-β (PeproTech, Cranbury, USA), 10 ng/mL IL-6 (PeproTech, Cranbury, USA) and 10 µg/mL anti-IFNγ (BioLegend, San Diego, USA) for Th17 differentiation [39]. MitoQ (1 µM, 6 h) were supplemented anterior to Th17 differentiation period.

### Western blotting

Western blots were performed using antibodies to PPAR-γ (Santa Cruz, Cat#SC-7273), β-actin (Sigma, Cat#A3854), and homemade antibodies against DsbA-L.

### Bioinformatic analysis

Publicly available data on acute colitis were downloaded from the Gene Expression Omnibus (GEO) database (GSE73424) (https://www.ncbi.nlm.nih.gov/geo/) [18]. After annotating the dataset with the R script in R 4.1.1 software (https://www.r-project.org/), we used the *“limma”* V3.44.3 package to screen DEGs in colon tissues between control and DSS acute colitis samples [40]. Significantly changed DEGs were identified using the cut-off thresholds of |logFC | >1 and *p* < 0.05. The volcano of DEGs was visualized by the *“ggplot2”* V3.3.5 package. In our study, KEGG analysis of DEGs was completed by *“clusterProfiler”* V3.16.0 package [41]. Gstk1 expression was trans-log2 transferred and compared by a two-tailed Student’s t-test. To further examine GSTK1 expression in human inflammatory bowel disease (IBD), publicly available datasets GSE179285 (ulcerative colitis, UC) and GSE66407 (Crohn’s disease, CD) were analyzed using GEO2R (https://www.ncbi.nlm.nih.gov/geo/geo2r/). Differential expression analysis of GSTK1 (encoding DsbA-L) in colonic biopsies revealed significant downregulation in both UC patients (Log2FC = -0.254, *P* = 0.00294; GSE179285) and CD patients (Log2FC = −0.108, *P* = 0.00823; GSE66407). Volcano plots were generated using SangerBox (http://sangerbox.com/), where red dots indicate significantly upregulated genes, green dots indicate significantly downregulated genes, and gray dots represent non-significant differentially expressed genes.

### qPCR analysis

Total RNAs were extracted using AG RNAex Pro Reagent (Accurate Biotechnology (Hunan) Co., Ltd, ChangSha, China). The cDNA synthesis and qPCR assay were performed as previously described [42]. The relative mRNA levels were calculated by the 2^-ΔΔCT^ method using β-Actin as an internal control. In qPCR data, samples were rarely excluded based on pre-established criteria: poor RNA quality or housekeeping gene expression differing by >2 Ct values from the sample average. The primer pairs used for qPCR were listed in Table S1.

### Randomization and blinding

Investigators were blinded to group allocation during data collection of histological specimens and metabolic tests. In experiments without subjective estimation, like flow cytometry and qPCR, investigators were unblinded during data collection since no bias would be introduced by the investigators. For all experiments, investigators were blinded during data analysis.

### Statistics

Statistical analyses were carried out using GraphPad Prism 8.0 Software (www.graphpad.com) or Microsoft Excel (Microsoft). Statistical parameters including the statistical test used, the exact value of n, what n represents, and measures of distribution and deviation are reported in the figure legends. Statistical analysis of the data was performed using analysis of variance (ANOVA) or Student’s *t*-test. Data shown are average ± standard error of the mean (SEM). The *p*-value of ≤0.05 was considered to be statistically significant.

## Supplementary information

primer and qPCR

Supplementary figure legends

Supp 1

Suppl 2

Suppl 3

Suppl 4

Original Data

## Data Availability

Gene expression profiling analysis was performed using publicly available datasets from the NCBI Gene Expression Omnibus (GEO; https://www.ncbi.nlm.nih.gov/geo/) database under accession numbers GSE73424, GSE179285, and GSE66407. The data that support the findings of this study were available from the corresponding author upon reasonable request.
