# Supplementary material for: DsbA-L safeguards T cell mitochondrial redox homeostasis to restrain Th17 differentiation and colitis
Source: Cell Death Dis. 2026 Jun 13;17(1):745. doi: 10.1038/s41419-026-08978-6 (PMC13490580; doi:10.1038/s41419-026-08978-6)
Supplement: Supplementary file 1 [file 41419_2026_8978_MOESM1_ESM.pdf]

Table S1 Nucleotide sequences of primers used for qPCR analysis

Mouse *β-Actin*	F: GATCATTGCTCCTCCTGAGC
	R: ACTCCTGCTTGCTGATCCAC
Mouse *Gstk1*	F: GTTGCGGCCCACTTTAATCG
	R: ATGTACTGGCCTTTTCGGGG
Mouse *Pparg*	F: ATTGAGTGCCGAGTCTGTGG
	R: GGCATTGTGAGACATCCCCA

Fig 5A

	Ctrl				Rosi			
Gstk1	1.000	0.955	0.789	0.800	2.290	1.862	1.760	2.290
Pparg	1.000	0.950	0.716	0.633	3.730	2.718	2.388	2.408
