# Supplementary material for: DsbA-L safeguards T cell mitochondrial redox homeostasis to restrain Th17 differentiation and colitis
Source: Cell Death Dis. 2026 Jun 13;17(1):745. doi: 10.1038/s41419-026-08978-6 (PMC13490580; doi:10.1038/s41419-026-08978-6)
Supplement: Supplementary file 2 [file 41419_2026_8978_MOESM7_ESM.pdf]

Figure1-F

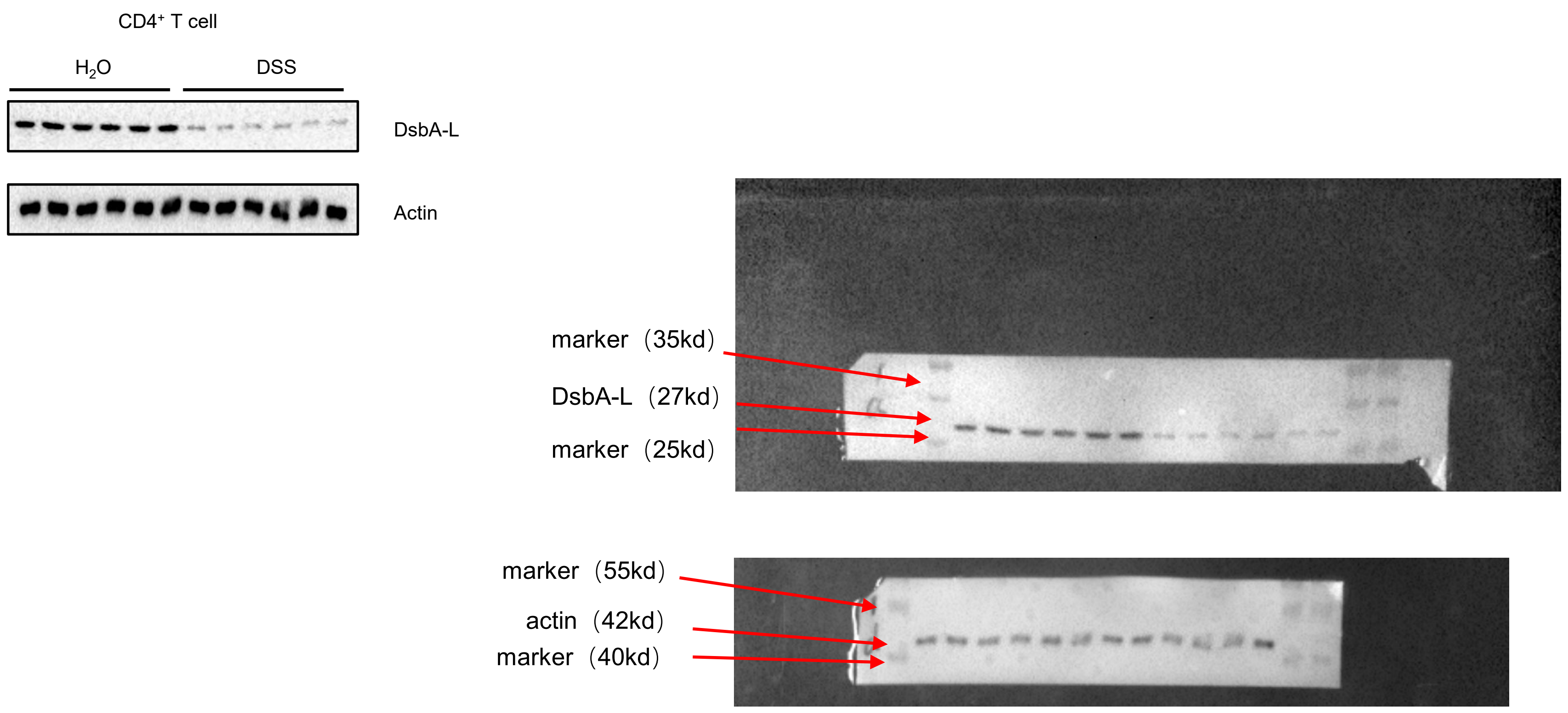

CD4+ T cell
H2O
DSS
DsbA-L
Actin
marker（35kd）
DsbA-L（27kd）
marker（25kd）
marker（55kd）
actin（42kd）
marker（40kd）

Figure5-B

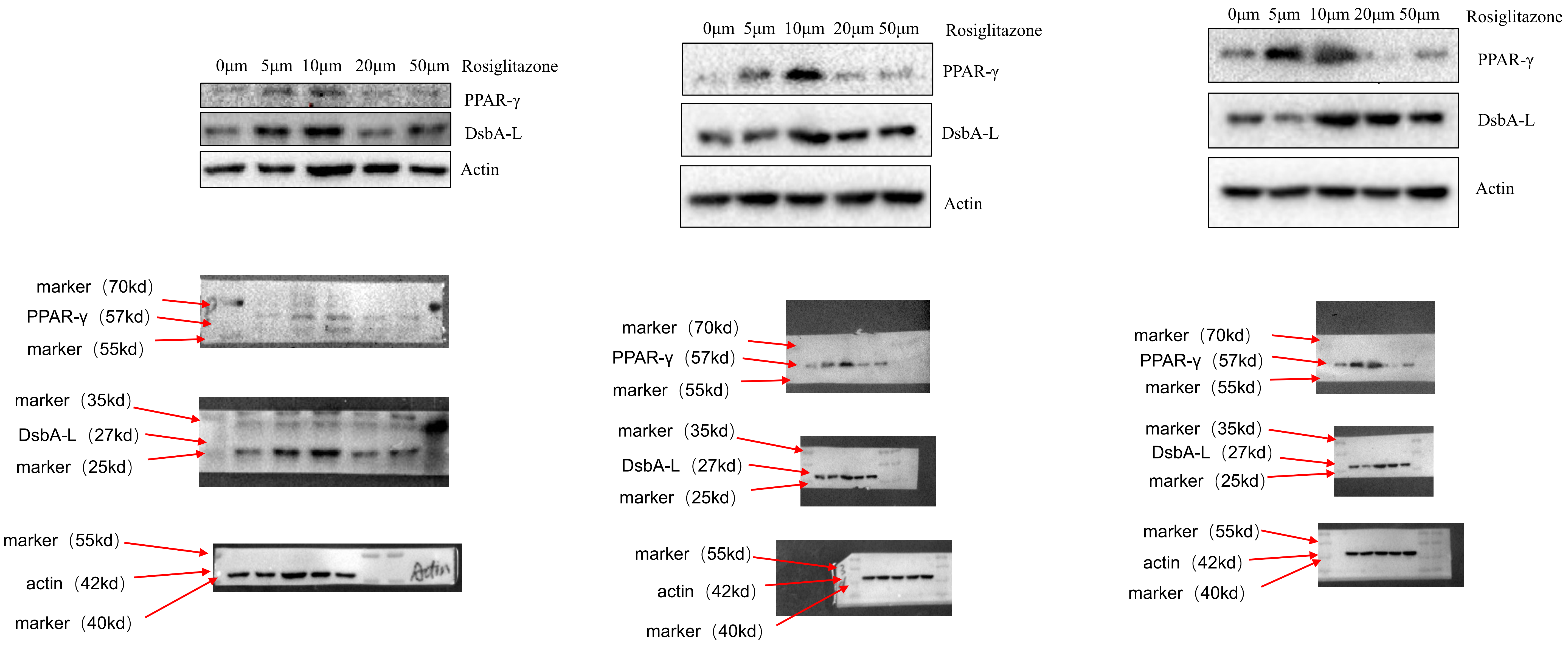

0μm 5μm 10μm 20μm 50μm Rosiglitazone
PPAR-γ
DsbA-L
Actin
0μm 5μm 10μm 20μm 50μm Rosiglitazone
PPAR-γ
DsbA-L
Actin
0μm 5μm 10μm 20μm 50μm Rosiglitazone
PPAR-γ
DsbA-L
Actin
marker（70kd）
PPAR-γ（57kd）
marker（55kd）
marker（35kd）
DsbA-L（27kd）
marker（25kd）
marker（55kd）
actin（42kd）
marker（40kd）
Actin
marker（70kd）
PPAR-γ（57kd）
marker（55kd）
marker（35kd）
DsbA-L（27kd）
marker（25kd）
marker（55kd）
actin（42kd）
marker（40kd）
marker（70kd）
PPAR-γ（57kd）
marker（55kd）
marker（35kd）
DsbA-L（27kd）
marker（25kd）
marker（55kd）
actin（42kd）
marker（40kd）
