# Supplementary material for: DsbA-L safeguards T cell mitochondrial redox homeostasis to restrain Th17 differentiation and colitis
Source: Cell Death Dis. 2026 Jun 13;17(1):745. doi: 10.1038/s41419-026-08978-6 (PMC13490580; doi:10.1038/s41419-026-08978-6)
Supplement: Supplementary file 3 [file 41419_2026_8978_MOESM2_ESM.docx]

**Figure legends**

**Suppl fig1. Gating strategy.** Gating strategy of mitochondrial mass (MitoTracker) and mtROS (MitoSOX) in CD4⁺ T cells from colonic lamia propria.

**Suppl fig2. DSS-induced colitis model established.** Weight change (A) and rectal bleeding (B) and colon length(C) in colonic lamina propria lymphocyte were detected from WT mice given 4% DSS for 6 days (n=4/group). (D) Kyoto Encyclopaedia of Genes and Genomes (KEGG) pathway analysis of DEGs. All data are presented as mean ± SEM. Significance were determined by two-way ANOVA or unpaired 2-tailed Student’s t test. * P < 0.05; ** P < 0.01.

**Suppl fig3. GSTK1 expression is downregulated in human IBD patients.** Volcano plots showing differential gene expression analysis of GSTK1 (encoding DsbA-L) in colonic biopsies from IBD patients versus healthy controls. (A) Ulcerative colitis (UC) patients (GSE179285) showing significant downregulation of GSTK1 (Log2FC = -0.254, P = 0.00294). (B) Crohn's disease (CD) patients (GSE66407) showing significant downregulation of GSTK1 (Log2FC = -0.108, P = 0.00823). Red dots indicate significantly upregulated genes, green dots indicate significantly downregulated genes, and gray dots represent non-significant differentially expressed genes.

**Suppl fig4. T cell subsets in spleen and mesenteric lymph nodes of DsbA-LCD4-KO mice after DSS challenging.** DsbA-LCD4-KO mice and control littermates were fed 4% DSS for 7 days (n=4-5/group). (A and B) Representative FACS plots of T cells subsets (left) and quantification of the frequencies of T cell subset (right) in spleen and mesenteric lymph nodes respectively. All data are presented as mean ± SEM. Significance was determined by unpaired 2-tailed Student’s t test.
